# The frequency of occurrence of Hepatocellular Carcinoma after direct antiviral therapy in Hepatitis C virus patients

**DOI:** 10.12669/pjms.35.1.109

**Published:** 2019

**Authors:** Bilal Aziz, Tazeen Nazar, Suhair Akhlaq

**Affiliations:** 1 Dr. Bilal Aziz, MBBS, MD(GI), FCPS(Med). Assistant Professor of Medicine, Mayo Hospital, Lahore, Pakistan; 2 Dr. Tazeen Nazar, MBBS, FCPS(Med). Assistant Professor of Medicine, Mayo Hospital, Lahore, Pakistan; 3 Suhair Akhlaq, MSc Statistics. Biostatistician, Ministry of Health and Prevention, Dubai, UAE

**Keywords:** Direct Acting Antiviral Agents, Hepatocellular carcinoma, Sustained Virological Response

## Abstract

**Objective:**

To study the frequency of occurrence of hepatocellular carcinoma (HCC) in hepatitis C virus patients treated with direct acting antiviral (DAA) agents.

**Methods:**

This hospital based cross-sectional study was conducted in Mayo Hospital, Lahore from June, 2016 to January, 2018. Total 300 patients with HCV genotype 3, selected via Non-Probability Purposive Sampling technique, without prior or concurrent history of HCC, were given DAA agents and were followed up for 6 months after completion of therapy. Results were based on Quantitative PCR to assess Sustained Virological Response (SVR) and Ultrasound Abdomen to look for the appearance of any new lesion. Data was presented as mean ±SD, frequency and percentages and was analyzed using SPSS Version 24.0.

**Results:**

Out of 300 patients, 179 (59.7%) were males and 121(40.3%) were females. Mean age of the patients was 55.08 ± 5.602 years. 214(71.3%) patients had compensated cirrhosis at the start of treatment and 86(28.7%) had decompensated cirrhosis. SVR was achieved in 200(93.4%) out of 214 patients with compensated cirrhosis and in 76(88.3%) out of 86 patients with decompensated cirrhosis. At six months post- treatment, 10(3.33%) patients developed HCC,2(0.7%) in the compensated group and 8(2.7%) in the decompensated group, out of which 5(6.6%) patients had achieved SVR.

**Conclusion:**

The frequency of HCC following DAA agents is significant (3.3%) even after achieving SVR. Caution must be exercised in prescribing DAA agents to HCV patients keeping this complication of HCC in mind.

## INTRODUCTION

Hepatocellular carcinoma (HCC) is one of the leading causes of death from cancer worldwide.^1^ In the United States, it has emerged to be the fastest growing cause of mortality in cancer patients.^2^ The presence of liver cirrhosis has been attributed to the development of hepatocellular carcinoma.^3^ Chronic infections with HBV and HCV leading to fibrosis have been identified as the major contributors of HCC.^4^ With the development of HBV vaccine, a significant decline in HBV related HCC cases has been observed^5^ but the cases related to HCV are on a rise. In a patient with HCV infection, it takes an average of 20-30 years for cirrhosis to occur, with HCC occurring in about 1-8% of these patients.^6^ Certain other factors that can have an important association with the risk of HCC include Non- Alcoholic Fatty Liver Disease (NAFLD) leading to Non- Alcoholic steatohepatitis (NASH)^7^ in addition to the viral load of HCV-RNA, age and sex of the patient, platelet counts and the duration of fibrosis.^8^

The presence and absence of cirrhosis is a major contributory factor to the risk of HCC. In HCV patients who do not have cirrhosis but have achieved Sustained Virological Response (SVR), the risk of developing HCC is lower compared to cirrhotic HCV patients, even after the attainment of SVR, the risk of HCC still persists. Although the incidence of HCV cases has shown a decline in the past few years but its prevalence in patients with HCC continues to rise.^9^

Over the past decade, the management of HCV infection has undergone a revolutionary change. Prior to that, interferon therapy emerged as a promising treatment option for this infection. In that era, not only that HCV infected cirrhotic patients who achieved SVR very rarely developed HCC but interferon therapy resulted in an improvement in the degree of fibrosis and even cirrhosis in HCV induced chronic liver disease.^10^ But the use of interferon therapy was restricted to patients with compensated liver disease only.

The Direct Acting Antiviral (DAA) agents have not only transformed the management of HCV infection but have also shown high efficacy in terms of achieving SVR in HCV patients. Their usefulness is further augmented by their role in improving liver function and reducing the need for liver transplantation in decompensated HCV infection.^11^,^12^

Several studies conducted worldwide, more so in the Western world, have not only shown the frequency of occurrence of new cases of HCC but have also demonstrated the recurrence of HCC in patients infected by HCV. Some studies have compared the occurrence of HCC in patients who received interferon based therapy with those who were given DAA agents. These studies have shown a marked variation in the results.

So far, very limited data is available for the Pakistani population demonstrating the frequency of HCC occurrence after treatment with DAA agents in patients with HCV genotype 3. The aim of this study was to determine the frequency of occurrence of HCC in HCV patients after successful treatment with DAA agents. We expected different results in our population because of the difference in genotype (as genotype 3 is more prevalent in the Pakistani population compared to Genotype-I in the Western world). Also the newer DAA drugs like Ledipasvir, ombitasvir / paritaprevir / ritonavir – dasabuvir and Valpatasvir are not readily available in the market so the patients were given only Sofosbuvir, Daclatasvir and Ribavirin.

## METHODS

This hospital based cross-sectional study was carried out in Mayo Hospital, Lahore from June, 2016 to January, 2018. Three hundred patients were selected via Non-Probability Purposive Sampling Technique, keeping 95% Confidence level, 3% margin of error and taking expected percentage of HCC in patients treated with DAA as 3.2%.^2^ Only those patients who did not have any prior or concurrent history of HCC, as assessed by a screening Ultrasound Abdomen at the time of enrollment, were selected for the study. The treatment regimen for the patients included DAA consisting of a Nucleotide analogue Sofosbuvir 400mg daily, NS5A inhibitor Daclatasvir 60mg daily with or without Ribavarin 800 mg daily. Only Child Pugh Class A and B were included in the study. The patients with compensated cirrhosis i.e., Child Pugh Class A were given only Sofosbuvir 400 mg along with Daclatasvir 60mg daily (double drug combination) for a period of 12 weeks. The patients with decompensated cirrhosis i.e., Child Pugh Class B at the time of enrollment were given a triple drug regimen (Sofosbuvir, Daclatasvir and Ribavarin) for 24 weeks. Baseline Complete Blood Counts (CBC), Liver Function Tests (LFTs), Serum Albumin, Prothrombin Time (PT), International Normalized Ratio (INR), Ultrasonography of the abdomen, Alpha Feto-protein (AFP), Anti HCV by ELISA followed by Quantitative PCR to detect viral load and HCV Genotyping were carried out. CBC, LFTs, Serum Albumin, PT, INR, Ultrasound abdomen were performed monthly till the duration of therapy and then 3-monthly for another six months. The most prevalent genotype in Pakistani population is genotype 3, so only genotype 3 patients were enrolled in the study. Both treatment-naive as well as treatment-experienced patients were enrolled in the study. Patients who had a suspicious lesion in the liver on ultrasound as well as those patients who had a co-infection with HBV or HIV were excluded from the study. Patients less than 18 and greater than 70 years were not included in the study. Pregnant females, extremely fragile and underweight individuals, patients with known psychiatric illness e.g., severe depression, or patients who were taking drugs like phenytoin, rifampin, carbamezepine etc, and patients with pancytopenia i.e., Hb <10g/dl, WBC <4 x10^3^, platelet < 100,000 were also excluded from the study. Quantitative PCR was also performed at the end of therapy with DAA agents and then at three months after the completion of therapy to assess Sustained Virological Response (SVR). Any suspicious lesion on the liver during or after the completion of therapy, as detected on Ultrasound Abdomen, was further confirmed by Serum AFP levels as well as Triphasic CT Scan of the Abdomen.

**Table-I:** Frequency distribution of all variables.

Variables	Male	Female	Average / Total
Age	53.89±5.56	56.83±5.20	55.08±5.62
Sex	179 (59.7%)	121(40.3%)	300
Child-Pugh class A	133(62.1%)	81(37.9%)	214 (71.3%)
Child-Pugh class B	46(53.5%)	40(46.5%)	86 (28.7%)
SVR Achieved	163(59%)	113(41%)	276 (92%)
SVR not achieved	16(66.6%)	8(33.3%)	24 (8%)
No HCC	173(59.7%)	117(40.3%)	290 (96.7%)
HCC	6(60%)	4(40%)	10 (3.3%)

All the collected data was entered and analyzed using computer software SPSS Version 24.0. Quantitative data like age, height, weight, BMI was calculated and presented as mean ±SD. Qualitative data like gender, presence or absence of HCC was presented in the form of frequency and percentages.

## RESULTS

A total of 300 patients were enrolled in the study, out of which 179 (59.7%) were males and 121(40.3%) were females. Mean age of the patients was 55.08± 5.62 years. Out of 179 males, 133 (74.3%) had a compensated cirrhosis at the start of treatment with DAA agents and 46 (25.6%) had decompensated cirrhosis. Likewise, 81 (67%) out of the 121 females enrolled in the study had compensated cirrhosis and 40 (33%) had decompensated cirrhosis. SVR was achieved in 200(93.4%) out of 214 patients with compensated cirrhosis (Child-Pugh class A), of which 123 (61.5%) were males and 77 (38.5%) were females, and in 76 (88.3%) out of 86 patients with decompensated cirrhosis (Child-Pugh class B), of which 40 (52.6%) were males and 36 (47.4%) were females. All these patients were then followed up for another three months after checking the status of SVR. In the compensated cirrhosis group, 2(14.3%) out of 14 patients who did not achieve SVR developed HCC at six months post-treatment. None of the patients with compensated cirrhosis who achieved SVR developed HCC. In the decompensated cirrhosis group, 8 (9.3%) out of 86 patients developed HCC, out of which 5(6.6%) out of 76 patients had achieved SVR and 3(30%) out of 10 patients had not achieved SVR. From a total of 10 patients who developed HCC, 6(60%) were males and 4(40%) were females. So this study showed a considerable percentage of patients who developed HCC i.e., 10 out of 300 patients (3.3%) following treatment with DAA agents for HCV genotype 3 infection.

**Table-II:** SVR and HCC in Child Pugh Class A and B.

Outcome	Child Pugh A (Compensated cirrhosis) n=214			Child Pugh B (Decompensated cirrhosis) n=86		

	Male	Female	Total	Male	Female	Total
SVR achieved	123	77	200(93.4%)	40	36	76(88.4%)
HCC in SVR achieved group	0	0	0	3	2	5(6.6%)
SVR not achieved	10	4	14(6.6%)	6	4	10(11.6%)
HCC in SVR not achieved group	2	0	2(14.3%)	2	1	3(30%)
Total HCC	2	0	2(14.3%)	5	3	8(9.3%)
No HCC	131	81	212(99%)	42	36	78(90.7%)

## DISCUSSION

HCV Genotype 3 is the second most prevalent genotype worldwide affecting approximately 54.3 million patients that account for 30.1%.^13^ Studies have been published in the last few years citing evidence that HCV genotype 3 is associated with an increase in the risk of progression to cirrhosis and to hepatocellular carcinoma. In a multivariate model analysis of the Swiss Hepatitis C Cohort Study, the various independent risk factors that were found to accelerate the progression to liver fibrosis included male sex [ OR= 1.60 (95% CI= 1.21-2.12), P <0.001], genotype 3 infection [OR= 1.89 (1.37-2.61), P <0.001], histological activity [ OR=2.03 (1.54-2.68), P< 0.001] and age at infection [OR= 1.08 (1.06-1.09), P< 0.001].^14^ Similarly, in a French cohort, a 34% occurrence rate of HCC was reported after 5 years in patients with HCV genotype 3 related cirrhosis.^15^

A study was conducted by L Renaldi and colleagues, on a population of 280 patients treated in four centers of southern Italy. The follow-up of patients in the first few months of therapy confirmed a high rate of SVR (97.1%). However, there were nine new cases of HCC in the early weeks following therapy with an incidence of 3.2%. Half of the HCC cases occurred at the end of the anti-viral treatment.^3^

Fasiha Kanwal along with her colleagues conducted a retrospective cohort study of HCV patients treated with DAA agents and calculated the annual incidence rates of HCC by SVR. Two hundred seventy one out of 22,500 patients developed HCC at the end of treatment, diagnosed during a follow up of 22963 PY. The annual incidence rate of HCC was found out to be 1.18 per 100 PY (or 1.18%, 95% CI, 1.04-1.32%). The annual incidence rate of 183 patients who achieved SVR during a follow up period of 20,415 PY was calculated to be 0.90 per 100 PY (or 0.90%, 95% CI, 0.77 - 1.03%). The patients who did not achieve SVR had a higher incidence rate of HCC i.e., 3.45 per 100 PY (or 3.45%, 95% CI, 2.73 - 4.18). The study concluded that those patients who were cirrhotic at the start of treatment had the highest annual incidence of HCC (1.82%, 95% CI, 1.52-2.12%vs 0.34%, 95% CI, 0.24-0.45% in patients who did not have cirrhosis). The calculated risk of HCC in cirrhotic patients was 4.7 times higher compared to non-cirrhotic patients (adjusted HR=4.73, 95% CI, 3.34-6.68).^16^

Studies have shown that patients with HCC who were successfully treated by various treatment modalities except liver transplantation, had a recurrence of their tumor after they achieved SVR with DAA agents. This was confirmed by a study carried out by Conti, et al on a group of 344 cirrhotic patients, out of which 59 patients had HCC previously and 285 did not have HCC. These patients were followed up for 24 weeks. Out of the 59 patients who previously had HCC, 17 had a recurrence whereas 9 out of 285 patients developed HCC who were previously tumor free.^17^ In these patients presence of advanced cirrhosis predicted the recurrence of HCC.

Another study was conducted by Reig M, et al. on a group of 103 patients successfully treated for HCC and were given DAA agents for HCV infection. The study was conducted between 2014 and 2015 and only 58 patients who fulfilled the inclusion criteria were followed up. The median follow-up time was 5.7 months. Mortality occurred in three patients and 16 patients had a recurrence of their tumor.^18^

## CONCLUSION

It was concluded that in the Decompensated Group, even after achieving SVR, a very significant (6.6%) proportion of patients developed HCC. Although at this stage, we cannot conclude that DAA agents have a direct influence on the occurrence of HCC. Also this study was conducted in only one tertiary care hospital on a small cohort of patients (300 only), large multi-centered trials need to be conducted to validate the results of this study. If proven significant, then caution must be exercised in the use of DAA agents particularly in Decompensated HCV patients.

### Authors’ Contribution

**BA** conceived and designed the study.

**TN** did data collection and manuscript writing.

**SA** did statistical analysis.

**BA and TN** edited, reviewed and gave final approval of the manuscript.
